# A polycomb-mediated epigenetic field defect precedes invasive cervical carcinoma

**DOI:** 10.18632/oncotarget.11390

**Published:** 2016-08-19

**Authors:** Neil Ari Wijetunga, Miriam Ben-Dayan, Jessica Tozour, Robert D. Burk, Nicolas F. Schlecht, Mark H. Einstein, John M. Greally

**Affiliations:** ^1^ Department of Genetics and Center for Epigenomics, Albert Einstein College of Medicine, Bronx, NY 10461, USA; ^2^ Department of Pediatrics (Genetics), Albert Einstein College of Medicine, Bronx, NY 10461, USA; ^3^ Department of Epidemiology and Population Health, Albert Einstein College of Medicine, Bronx, NY 10461, USA; ^4^ Department of Medicine (Oncology), Albert Einstein College of Medicine, Bronx, NY 10461, USA; ^5^ Department of Obstetrics & Gynecology and Women's Health, Albert Einstein College of Medicine, Bronx, NY 10461, USA

**Keywords:** cervical carcinoma, polycomb, DNA methylation, field defect, epigenetic

## Abstract

Human papillomavirus (HPV)-associated cervical carcinoma is preceded by stages of cervical intra-epithelial neoplasia (CIN) that can variably progress to malignancy. Understanding the different molecular processes involved in the progression of pre-malignant CIN is critical to the development of improved predictive and interventional capabilities. We tested the role of regulators of transcription in both the development and the progression of HPV-associated CIN, performing the most comprehensive genomic survey to date of DNA methylation in HPV-associated cervical neoplasia, testing ~2 million loci throughout the human genome in biopsies from 78 HPV+ women, identifying changes starting in early CIN and maintained through carcinogenesis. We identified loci at which DNA methylation is consistently altered, beginning early in the course of neoplastic disease and progressing with disease advancement. While the loss of DNA methylation occurs mostly at intergenic regions, acquisition of DNA methylation is at sites involved in transcriptional regulation, with strong enrichment for targets of polycomb repression. Using an independent cohort from The Cancer Genome Atlas, we validated the loci with increased DNA methylation and found that these regulatory changes were associated with locally decreased gene expression. Secondary validation using immunohistochemistry showed that the progression of neoplasia was associated with increasing polycomb protein expression specifically in the cervical epithelium. We find that perturbations of genomic regulatory processes occur early and persist in cervical carcinoma. The results indicate a polycomb-mediated epigenetic field defect in cervical neoplasia that may represent a target for early, topical interventions using polycomb inhibitors.

## INTRODUCTION

Despite the effectiveness of screening programs for cervical cancer in developed countries, it remains the third most common cancer in women, affecting 500,000 worldwide each year, leading to 275,000 deaths in 2008 [1]. Human papillomavirus (HPV) is necessary but is not sufficient to cause cervical cancer [2]. It is not known what additional immunologic, genetic, and molecular mechanisms are involved in malignant transformation. There is a well-defined natural history of progression from normal cervical epithelium to the histological appearance of low-grade cervical intraepithelial neoplasia (CIN), high grade CIN, and cancer. However, only a minority of women presenting with the lower grade CIN1 stage progress to the more advanced CIN3, and a further minority of women with CIN3 progress to cervical cancer [3]. Most women with precancerous disease have spontaneous regression of lesions, prompting the need for tests that allow the accurate prediction of the subset of women at risk of progression, who require more aggressive intervention to manage their disease. At present, the dual immunostaining of p16(INK4a)/Ki-67 in addition to studies of viral methylation and integration appear to have potential as molecular biomarkers, whereas candidates from studies of DNA methylation have either shown inconsistent results or have yet to be validated in prospective studies [4, 5]. There therefore remains a significant need for improved insights into the ability to identify women at greatest risk of disease progression [6]. Furthermore, given the relationship between extirpative cervical procedures and preterm birth [7, 8], there is a need for non-surgical selective targeted therapies.

Abnormal regulation of gene expression has been studied as a possible complementary mechanism of development of cervical carcinoma [9–13]. HPV proteins E6 and E7 are known to have effects to induce DNA methyltransferases [14, 15] while E6 is also found to induce the lysine demethylases KDM6A and KDM6B, which normally remove methyl groups from histone H3 lysine 27 [16]. Prior genome-wide studies of DNA methylation testing~27,000 loci throughout the genome has been performed, testing histologically-normal exfoliated cells from women who subsequently developed CIN2 [17, 18], and exfoliated samples of CIN2 or cervical carcinoma biopsies with matched exfoliated control samples [18]. The studies of epithelium destined for dysplastic changes showed increased variability of DNA methylation [17], with acquisition of DNA methylation at loci known to be targeted by polycomb repressive complex during development [17, 18]. *In vitro* studies of cervical carcinoma cell lines with DNA methyltransferase inhibitors have shown reversal of silencing of genes and restoration of sensitivity to chemotherapeutic agents [19–21].

The current study builds upon these prior studies by performing genome-wide DNA methylation studies on biopsies from 78 samples from women across the progressive stages of cervical carcinoma. Our study design is cross-sectional, but used samples collected from women followed up over time, allowing us to focus on individuals with early stage (CIN1) disease that persisted rather than resolved spontaneously. The availability of data from The Cancer Genome Atlas (TCGA, http://cancergenome.nih.gov) allowed us to validate our findings in an independent cohort, leading us to studies of polycomb expression that provide support for an epigenetic field defect in the cervical epithelium of women with CIN who progress to develop cervical cancer.

## RESULTS

### Genome-wide DNA methylation changes during cervical cancer progression

**Figure 1:**
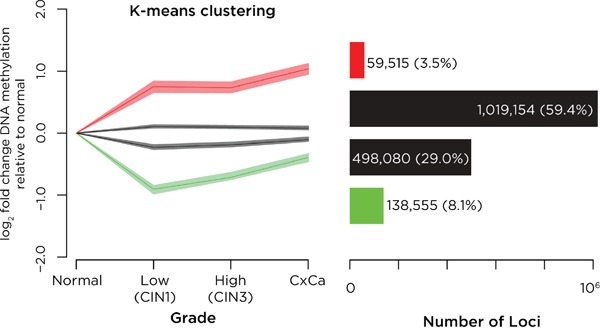
Analysis of DNA methylation changes with progression of cervical neoplasia K-means clustering was performed on all sites tested, representing the four groups in the left panel by showing the degree of change in DNA methylation relative to the value in normal epithelium, and showing for each group the mean and the broader 95% confidence interval (defined using bootstrapping) of values of DNA methylation. On the right we show the numbers of loci in each group. We find that most loci have minimal changes in DNA methylation (black) but that there exist small subgroups of loci defined by k-means clustering that gain (red) or lose (green) DNA methylation with disease progression.

We describe our patient and sample characteristics in Supplementary Table S1. We tested DNA methylation in biopsies of normal cervix, persistent CIN1 (low grade CIN), CIN2/3 (high grade CIN) and cervical cancer (CxCa). We used a K-means clustering approach to study these data, finding that four clusters were optimal to describe the distinctive patterns of DNA methylation associated with increasing disease grade (Supplementary Figure S2a). This approach revealed subsets of loci with increasing (n=59,515) and decreasing DNA methylation trends (n=138,555) (Figure 1).

**Figure 2:**
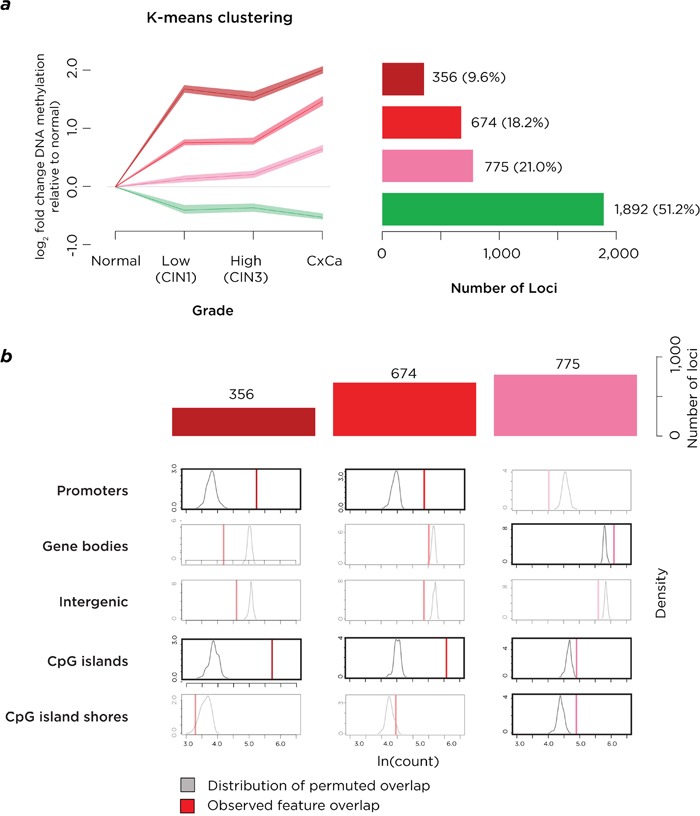
Analysis of loci with high-confidence changes of DNA methylation **a.** K-means clustering on this subgroup of loci again shows a group of loci to lose DNA methylation (green) but also shows three sets of loci gaining DNA methylation early (maroon), progressively (red) or late (pink) in disease progression. The numbers of loci in each group are represented on the right of the panel. **b.** When the three groups of loci gaining DNA methylation are tested, they are found to be enriched at CpG islands, with the earlier changes also at RefSeq gene promoters and late acquisition also occurring at CpG island shores. The acquisition of DNA methylation is therefore targeting loci with transcriptional regulatory properties.

We refined the analysis to focus on a subset of loci by using covariate-adjusted polytomous regression modeling, filtering to high-confidence significant loci with increasing (n=1,810) or decreasing (n=1,887) DNA methylation during disease progression. A further K-means clustering analysis was performed on 4 groups (Supplementary Figure S2b), leading to the loci with increasing DNA methylation being divided into 3 sub-clusters, with early (n=356), progressive (n=674) and late (n=775) acquisition of DNA methylation (Figure 2a). Permutation analyses revealed that early and progressive acquisition of DNA methylation was targeted to RefSeq gene promoters and CpG islands, and late acquisition of DNA methylation was also targeted to CpG island shores (Figure 2b). Loci with decreasing DNA methylation were enriched at intergenic sequences and CpG island shores but not promoters or CpG islands (Supplementary Figure S3). The gain of DNA methylation during progression of cervical neoplasia was therefore distinctive for targeting candidate *cis*-regulatory loci. We also combine the DNA methylation, disease stages and K-means clustering results in a single visualization to illustrate the relationships between these findings (Supplementary Figure S4).

### Properties of genes targeted by dysregulation of DNA methylation

**Figure 3:**
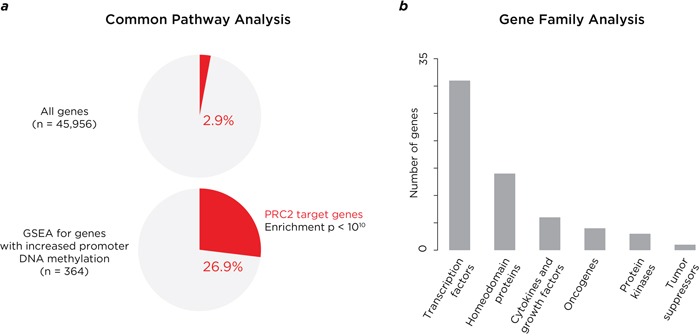
Analysis of properties of genes where DNA methylation is acquired at promoter-proximal locations The genes with promoter-proximal acquisition of DNA methylation with disease progression are striking for representing known targets for polycomb repressive complex 2 (PRC2), as shown in panel **a.**, which compares the proportion of PRC2 target genes throughout the genome (2.9%) and those with increased promoter methylation (26.9%). In panel **b.** we show the properties of the gene familes at which these changes are taking place, with enrichment especially for genes encoding proteins with transcriptional regulatory properties (transcription factors and homeodomain proteins).

To gain insights into functional consequences of these altered DNA methylation patterns in pre-malignant cells, we identified 457 gene promoters where DNA methylation increased with the early, progressive and late patterns. GSEA revealed a significant (p<10^−10^) representation of genes known to be targets of polycomb-mediated repression (Figure 3a, Supplementary Table S2), where promoters had distinctively high CG dinucleotide content (Supplementary Figure S5). Enrichment was also found for genes encoding transcription factors and homeodomain proteins (Figure 3b, Supplementary Table S3).

### Verification and validation of DNA methylation data

**Figure 4:**
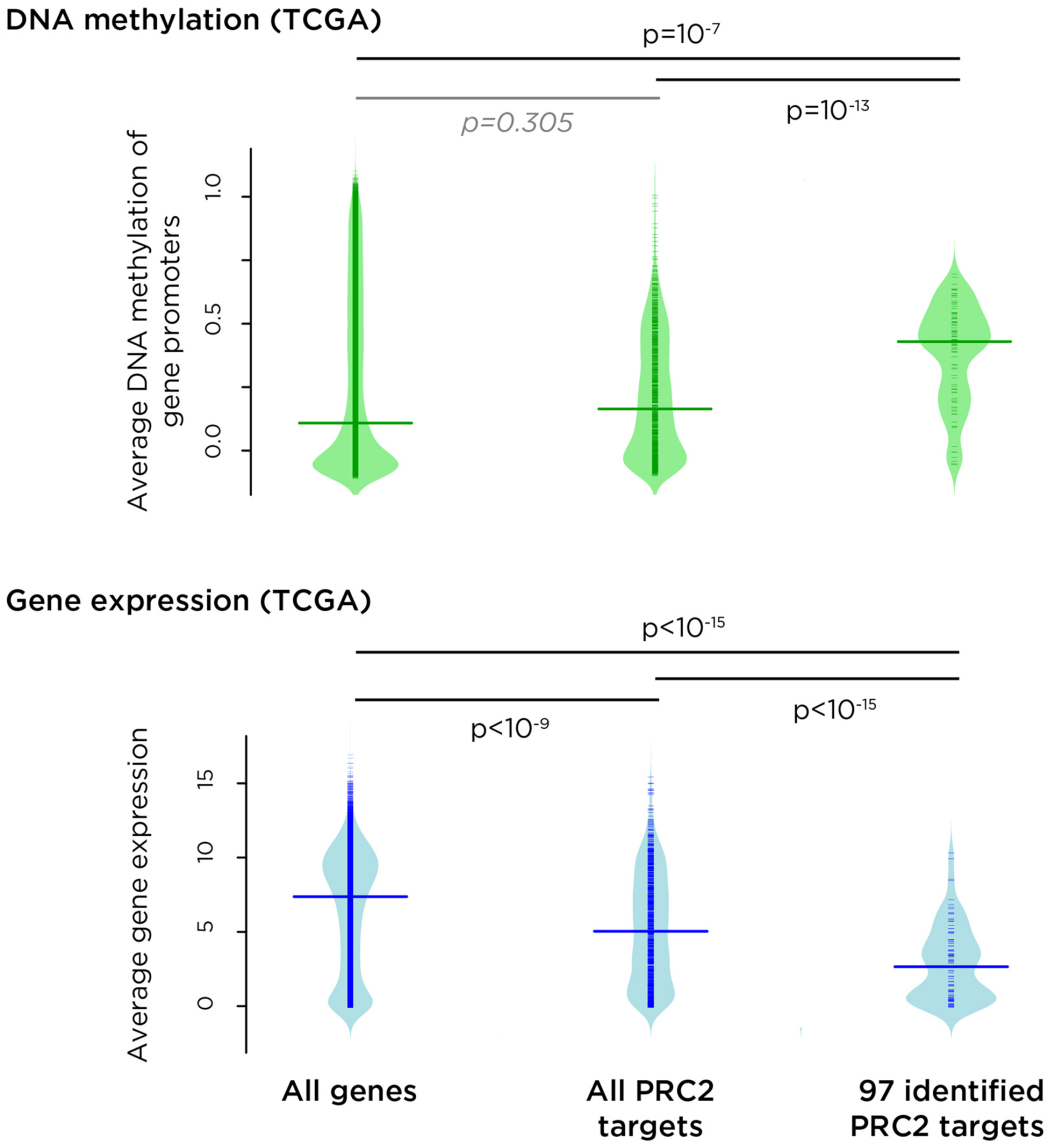
Independent validation of the results of this study using data from The Cancer Genome Atlas (TCGA) For DNA methylation (top, green) and RNA-seq (bottom. blue), we separately plotted the density of signal for all genes, all PRC2 target genes and the subset of PRC2 genes implicated by k-means clustering to have early, progressive promoter DNA methylation. We find that PRC2 target genes, especially those where we find DNA methylation acquisition with disease progression, are targeted for increased DNA methylation in TCGA subjects, and that those genes have a corresponding decrease in expression levels (p values shown).

The HELP-tagging data were verified using large-scale targeted bisulphite sequencing (Supplementary Figure S6, Supplementary Table S4). To validate the results with completely independent data, results of studies of a cervical cancer cohort from TCGA were used. The TCGA data revealed increased promoter DNA methylation at the 97 polycomb repressive complex 2 (PRC2) target genes that we found to have increased promoter DNA methylation in our high-grade disease and cancer cases (Figure 4a). TCGA gene expression data demonstrated that that these genes have significantly repressed expression (Figure 4b). We also found that PRC2 target genes are preferential targets for mutations in cervical cancer (Supplementary Figure S7).

### Immunohistochemistry of cervical cancer and precursor lesions

**Figure 5:**
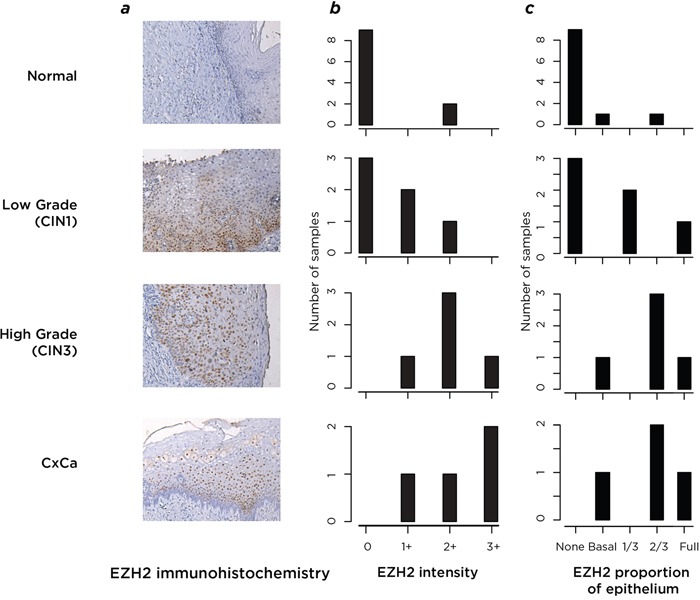
Analysis by immunohistochemistry of the expression of the polycomb protein EZH2 In **a.**, we show representative results of the immunohistochemistry images. Normal epithelium (top) was compared with the CIN1-grade areas within samples from all stages of disease progression, testing the epithelial “field” in which the more advanced lesions developed. These samples were assessed by a pathologist who created a scoring system of EZH2 signal intensity and proportional expression within the epithelium. In **b.** we plot this quantification of EZH2 signal intensity, and in **c.** the proportion of epithelium observed to be stained by EZH2. From the results of (b) and (c) it can be seen that both the intensity and the epithelial proportion in which EZH2 is expressed increases with disease progression (proceeding from top to bottom of image), with minimal expression of EZH2 found in normal epithelium.

To test whether the DNA methylation and polycomb effects could be attributed to the epithelial component of the cervical biopsy tissue, we performed immunohistochemistry on a subset of 15 samples, testing the EZH2 component of the polycomb complex. We show in Figure 5 that the EZH2 protein is usually not detectably expressed in normal cervical epithelium, but in samples of abnormal epithelium from progressive stages of cervical neoplasia it is robustly expressed in cells of epithelial origin. To make the samples as comparable as possible across disease stages, we studied regions within all of the biopsies where early grade neoplastic changes were found, to reduce the influence of DNA mutations in later stage disease having effects on EZH2 and to test whether the polycomb changes were occurring physically beyond the later-stage tumor itself. EZH2 was substantially upregulated in all neoplastic samples compared with normal cervical epithelium, with a trend towards more consistent, abundant and pervasive expression in surrounding epithelium as disease progresses. These results support the observed regulatory changes to be present within the epithelial cell component of the cervical biopsies, and not an unrecognized cell subtype effect that can confound DNA methylation studies [22].

## DISCUSSION

This study is the most comprehensive genomic regulatory profiling to date of the progressive stages of cervical carcinoma. Unusually among cancers, cervical carcinoma has a well-documented natural history that permits testing of recognizable precursor states, allowing us to identify pre-malignant molecular events in the cervical epithelial cells. Our findings show that DNA methylation is acquired at *cis*-regulatory sites in the genome at the earliest recognizable stage of neoplasia (persistent CIN1), targeting loci known to be polycomb targets, with associated increases in polycomb protein expression as a concurrently early event. Furthermore, the similar findings in the independent TCGA data indicate the robustness of our observations across independent cohorts. The results suggest the cervical epithelium to undergo a ‘field defect’ involving the acquisition of DNA methylation and polycomb-mediated repression, making the cervical cells susceptible to the subsequent effects of mutations that drive growth of cells within an epigenetically dysregulated field of cervical epithelium. Our findings support and extend those of prior studies based on exfoliated rather than biopsy samples of premalignant lesions, implicating acquisition of DNA methylation at developmental targets of polycomb repressive complex as an early and persistent event in cervical neoplasia [17, 18].

The relationship between DNA methylation and polycomb-mediated gene silencing is complex, initially believed to be a simple co-repression based on the association of DNA methyltransferases with polycomb group protein complexes [23], but later found to have more complex genomic associations, revealed by bisulphite sequencing of DNA from chromatin with polycomb-mediated histone H3 lysine 27 trimethylation (H3K27me3), both studies showing co-localization of H3K27me3 and DNA methylation throughout the genome except at CpG islands [24, 25], and one showing further perturbations of the association in neoplastic cells [25]. For HPV-associated neoplasia, the molecular mechanism of perturbation of transcriptional regulators may involve direct effects by the HPV itself. The E7 protein in HPV16 has been found to induce *EZH2* expression by releasing E2F from pocket proteins like pRb [26]. The long non-coding RNA (lncRNA) *HOTAIR* has been found to recruit the PRC2 polycomb group complex [27], and has been found to be a target for E7 [28], suggesting that lncRNAs (such as lncRNA-EBIC [29]) may be involved in targeting E7-induced polycomb effects in the genome.

As a prognostic biomarker of cervical carcinoma, the expression of EZH2 has been previously found to be informative. Consistent with our results, EZH2 was found to be rarely expressed in normal cervical epithelium but increasingly with disease stage progression [30]. This and a later study [31] revealed increased EZH2 expression to be associated with more severe cervical carcinoma, the later study also finding p53 expression to be associated with more advanced cancers. DNA methylation has also been shown to have value as a prognostic indicator in candidate gene studies [5, 10, 12]. Our genome-wide survey of DNA methylation reveals its early acquisition at a number of loci, defining a novel panel of candidate loci that can be tested in prognostic studies of cervical neoplasia, potentially in combination with expression studies of EZH2 and other informative proteins, to improve precision upon current clinically-used prognostic tests such as p16/Ki-67.

We recognize that there are limitations to our study design. We tested varying degrees of CIN and cancer, including what we would consider clinically relevant disease; persistent CIN1, high-grade CIN2/3, and CxCa. However, given that we treat all high-grade CIN and cancer, the development of biomarkers for these groups is of less value than for the preceding CIN1 stage, which is therefore the practical focus of the study. Moreover, we would need a substantially larger study sample size to adjust for potential confounding effects of immune status, HPV genotype and other putative factors that could potentially influence DNA methylation. Additionally, we employed only one secondary validation test, p16/Ki-67, which, while widely used, could be reinforced by additional validation studies.

The implication of epigenetic dysregulatory events in an epithelial tumor like cervical neoplasia offers a strategy for intervention. Both EZH2 [32] and DNA methyltransferases [33] can be targeted pharmacologically, potentially allowing reversal of the acquisition of DNA methylation and polycomb effects in the pre-malignant stages. The use of the EZH2 inhibitor GSK343 on the cervical carcinoma cell lines HeLa and SiHa *in vitro* was found to reduce cell proliferation, motility and invasiveness [34]. The pharmacokinetics of GSK343 in rodents show rapid clearance [32], so the toxicity *in vivo* of the agent is unknown, while the *in vivo* use of DNA methyltransferase inhibitors is recognizably associated with toxicity [33]. It is therefore possible that existing agents targeting transcriptional regulators could be adopted for similar interventions in the early, pre-malignant stages of cervical cancer, perhaps by topical administration to limit systemic toxicity, with the goal of reversing the local epigenetic field defect.

## MATERIALS AND METHODS

### Samples

We obtained cervical biopsies from 78 HIV-seropositive and negative patients attending gynecology clinics at the Montefiore Medical and North Bronx Hospital Centers in the Bronx (NY), corresponding to normal cervix (19 subjects), persistent low grade cervical intraepithelial neoplasia (CIN1, 20 subjects), high grade CIN2 or 3 (16 subjects), and invasive cervical carcinoma (CxCa, 23 subjects). CD4+T-cell counts and HIV viral load levels in the HIV-seropositive patients assessed within six-months of their CIN/cervical cancer diagnosis were abstracted from the electronic medical records.

Patients with low grade CIN1 were sequentially and serially biopsied for 6 months to define a group of women with disease persistence. All 78 biopsies were taken from women who were at least 18 years old. The procedures followed were in accordance with the ethical standards of the responsible committee on human experimentation (institutional or regional) or with the Helsinki Declaration of 1975, as revised in 1983. This study was approved by the institutional review board (IRB) at the Albert Einstein College of Medicine and is in accordance with Health Insurance Portability and Accountability Act (HIPAA) regulations. Written informed consent was obtained from all subjects prior to participation.

### HPV DNA genotyping

DNA extracted from liquid-based cytology smears was digested with proteinase-K/Laureth-12, precipitated and purified in ethanol, and amplified by PCR with Gold-Taq using a well-described the MY09/MY11 protocol [35, 36], followed by Southern blot hybridization with generic probes for HPV and an oligonucleotide for human β-globin DNA (as a control). PCR products positive by Southern blot were analyzed using biotinylated type-specific oligonucleotide probes for >40 different HPV types, including high-risk types (types 16, 18, 31, 33, 35, 39, 45, 51, 52, 56, 58 and 59) [37]. Samples that tested positive by the generic probe mix but negative by all type-specific probes were considered to represent “uncharacterized” HPV types.

### DNA methylation assays and analyses

For all 78 cervical biopsies (Supplementary Table S1), we extracted genomic DNA from a component of the frozen biopsy. We used this DNA to prepare libraries for the HELP-tagging assay [38], a massively parallel sequencing-based assay to study DNA methylation at ~2 million HpaII sites throughout the human genome, providing better representation of *cis*-regulatory elements than other commonly-used survey assays of DNA methylation [39]. To minimize potential batch effects, HELP-tagging libraries were processed in groups that were balanced by disease grade, HIV status, and patient age (±10 years). Each library was sequenced on the Illumina HiSeq 2000 platform. We used our previously described analytical approach [38, 40] to generate a measure of DNA methylation at each site tested, based on HpaII/MspI ratios that are approximately Cauchy distributed, so the cumulative distribution function (CDF) of the Cauchy distribution was used to obtain DNA methylation probabilities. Finally, mixture modeling of the distribution assuming three composite distributions was performed to scale the probabilities between 0 and 1. Assayed CpG sites with no reads in the HpaII channel and less than 5 reads in the MspI channel were removed from the analysis as loci in which we had less confidence in the accuracy of the DNA methylation estimations.

### Identifying and characterizing loci with significant changes of DNA methylation

To generate high-confidence associations between DNA methylation and the cervical neoplasia disease grades, we used principal components analysis (PCA) to identify biological and technical covariates that were associated with the variability in DNA methylation (Supplementary Figure S1). Covariates found to be associated with DNA methylation to a greater extent than disease grade (e.g., detection of high-risk HPV DNA and level of HIV control) were controlled for in downstream analyses by inclusion in linear models to obtain covariate-adjusted estimates. For robust data analysis and visualization, we employed batch correction implemented through the *ComBat* package in R [41] to obtain DNA methylation values adjusted for sequencing batch and control status of HIV co-infection. We performed polytomous regression, a modeling technique that is suited for analyzing DNA methylation proportions and allows simultaneous assessment of the odds of methylation at disease grade relative to normal, in R using the *multinom* function available through the *nnet* package. The odds of DNA methylation at each disease grade relative to normal cervix were calculated, adjusting for the sequencing batch and the HIV control. Overall model significance was assessed by adjustment for multiple comparisons using a false discovery rate (FDR) <0.05, and two criteria were used for further determination of model significance: (a) CxCa compared to normal cervix methylation effect significance <0.05 and (b) an average methylation difference between CxCa and normal cervix of 10%.

We explored where the significant DNA methylation changes were occurring relative to genomic annotations. We tested RefSeq gene promoters (the 2 kb region flanking the gene transcription start site (TSS)), the remaining gene body and intergenic regions, and CpG islands and their 2 kb flanking region (shores [42]). We measured enrichment by comparing the observed overlap between loci of interest and a particular annotation with the expected overlap given the total coverage of the annotation. We tested the significance of these enrichments using permutation tests, comparing the observed overlap of *n* loci of interest with a particular annotation *X* with 100 samples of *n* loci from all HpaII sites tested 100 times, comparing the observed frequency of *X* with the distribution of simulated annotation overlaps.

### K-means clustering

To study the dynamic changes of DNA methylation with progression of the disease, we used K-means clustering, an unsupervised clustering approach, using the *kmeans* function in R. We determined an optimal number of clusters by plotting the total within cluster sums of squares against the number of clusters and selecting a cluster value that occurred at the first inflection point.

### Gene set enrichment analysis (GSEA)

Using the GSEA [43] tool from the Broad Institute, we were able to cross-reference our identified genes of interest to a database of common pathways, the Molecular Signatures Database v 4.0 (MSigDB). The MSigDB contains curated gene sets assembled from previous studies. The overlap between our genes on interest and known gene pathways was analyzed using a hypergeometric distribution with a false discovery rate used to correct for multiple hypothesis testing.

### Verification of DNA methylation results

We performed large scale targeted bisulphite sequencing to verify DNA methylation results from 66 individuals (18 control, 19 CIN1, 11 CIN3 and 18 CxCa) at 70 different loci. These HpaII loci were chosen to test the full range of HELP-tagging values in each of the four disease groups. Using the UCSF *MethPrimer* tool, we designed primers to amplify bisulphite-converted target DNA sequences. *BiSearch* was used to avoid primer sets that generate off-target amplicons. Bisulphite treated DNA was pre-amplified with an equimolar primer mix, then unique dual indexed adapters were added and libraries amplified using a Fluidigm Access Array. The resulting amplicon library was sequenced using 150 bp paired end sequencing with the Illumina MiSeq platform. Reads were aligned to the human genome and DNA methylation ratios were calculated using *BSMAP 2.7.3*.

### Validation using the cancer genome atlas data

Using The Cancer Genome Atlas (TCGA) datasets for cervical squamous cell carcinoma and endocervical adenocarcinoma, we identified 196 samples with Cervical Squamous Cell Carcinoma, for which 183 samples had DNA methylation tested using Infinium HumanMethylation450 Methylation arrays, 192 had RNA-seq gene expression and 155 had somatic mutation data available. We associated DNA methylation at sites located ±2 kb from the RefSeq TSS with that gene, while RNA-seq data were already associated with genes. For DNA methylation and RNA-seq, we plotted the density of signal for all genes, all PRC2 target genes and the subset of PRC2 genes implicated by K-means clustering to have early, progressive promoter DNA methylation. For somatic mutation data, we plotted the density of the number of samples that contained at least one nonsense mutation occurring in all genes, all PRC2 target genes and all PRC2 genes implicated as having early, progressive promoter DNA methylation.

### Immunohistochemistry

We used immunohistochemistry (IHC) on 15 of the primary samples used for our DNA methylation studies to test expression of the candidate EZH2 protein implicated by our DNA methylation studies. We identified regions within each biopsy in which there was an area of low grade CIN and, when possible, an area of normal epithelium, and compared between samples the presence of EZH2, its expression location within the epithelial layers and the level of expression per cell. A relative scoring system was used for EZH2 signal intensity, and the proportion of epithelium with EZH2 signal was also assessed by the pathologist.

### Data availability

All sequencing data generated in this study are deposited at the Gene Expression Omnibus and are available in Series GSE76986.

## SUPPLEMENTARY FIGURES AND TABLES







## References

[1] Schiffman M, Castle PE, Jeronimo J, Rodriguez AC, Wacholder S (2007). Human papillomavirus and cervical cancer. The Lancet.

[2] de Sanjose S, Quint WG, Alemany L, Geraets DT, Klaustermeier JE, Lloveras B, Tous S, Felix A, Bravo LE, Shin HR, Vallejos CS, de Ruiz PA, Lima MA (2010). Human papillomavirus genotype attribution in invasive cervical cancer: a retrospective cross-sectional worldwide study. Lancet Oncol.

[3] Schiffman M, Wentzensen N (2013). Human papillomavirus infection and the multistage carcinogenesis of cervical cancer. Cancer Epidemiol Biomarkers Prev.

[4] Litjens RJ, Hopman AH, van de Vijver KK, Ramaekers FC, Kruitwagen RF, Kruse AJ (2013). Molecular biomarkers in cervical cancer diagnosis: a critical appraisal. Expert Opin Med Diagn.

[5] Wentzensen N, Sherman ME, Schiffman M, Wang SS (2009). Utility of methylation markers in cervical cancer early detection: appraisal of the state-of-the-science. Gynecol Oncol.

[6] Ikenberg H, Bergeron C, Schmidt D, Griesser H, Alameda F, Angeloni C, Bogers J, Dachez R, Denton K, Hariri J, Keller T, von Knebel Doeberitz M, Neumann HH (2013). Screening for cervical cancer precursors with p16/Ki-67 dual-stained cytology: results of the PALMS study. J Natl Cancer Inst.

[7] Pinborg A, Ortoft G, Loft A, Rasmussen SC, Ingerslev HJ (2014). Cervical conization doubles the risk of preterm and very preterm birth in assisted reproductive technology twin pregnancies. Hum Reprod.

[8] Sadler L, Saftlas A, Wang W, Exeter M, Whittaker J, McCowan L (2004). Treatment for cervical intraepithelial neoplasia and risk of preterm delivery. JAMA.

[9] Farkas SA, Milutin-Gašperov N, Grce M, Nilsson TK (2013). Genome-wide DNA methylation assay reveals novel candidate biomarker genes in cervical cancer. Epigenetics.

[10] Siegel EM, Riggs BM, Delmas AL, Koch A, Hakam A, Brown KD (2015). Quantitative DNA methylation analysis of candidate genes in cervical cancer. PLoS ONE.

[11] Wang KH, Lin CJ, Liu CJ, Liu DW, Huang RL, Ding DC, Weng CF, Chu TY (2015). Global methylation silencing of clustered proto-cadherin genes in cervical cancer: serving as diagnostic markers comparable to HPV. Cancer Med.

[12] Vasiljević N, Scibior-Bentkowska D, Brentnall AR, Cuzick J, Lorincz AT (2014). Credentialing of DNA methylation assays for human genes as diagnostic biomarkers of cervical intraepithelial neoplasia in high-risk HPV positive women. Gynecol Oncol.

[13] Louvanto K, Franco EL, Ramanakumar AV, Vasiljević N, Scibior-Bentkowska D, Koushik A, Cuzick J, Coutlée F, Lorincz AT (2014). Biomarkers of Cervical Cancer Risk Study Team. Methylation of viral and host genes and severity of cervical lesions associated with human papillomavirus type 16. Int J Cancer.

[14] Au Yeung CL, Tsang WP, Tsang TY, Co NN, Yau PL, Kwok TT (2010). HPV-16 E6 upregulation of DNMT1 through repression of tumor suppressor p53. Oncol Rep.

[15] Burgers WA, Blanchon L, Pradhan S, de Launoit Y, Kouzarides T, Fuks F (2007). Viral oncoproteins target the DNA methyltransferases. Oncogene.

[16] McLaughlin-Drubin ME, Crum CP, Münger K (2011). Human papillomavirus E7 oncoprotein induces KDM6A and KDM6B histone demethylase expression and causes epigenetic reprogramming. Proc Natl Acad Sci U S A.

[17] Teschendorff AE, Jones A, Fiegl H, Sargent A, Zhuang JJ, Kitchener HC, Widschwendter M (2012). Epigenetic variability in cells of normal cytology is associated with the risk of future morphological transformation. Genome Med.

[18] Zhuang J, Jones A, Lee SH, Ng E, Fiegl H, Zikan M, Cibula D, Sargent A, Salvesen HB, Jacobs IJ, Kitchener HC, Teschendorff AE, Widschwendter M (2012). The dynamics and prognostic potential of DNA methylation changes at stem cell gene loci in women's cancer. PLoS Genet.

[19] Huang Y, Song H, Hu H, Cui L, You C, Huang L (2012). Trichosanthin inhibits DNA methyltransferase and restores methylation-silenced gene expression in human cervical cancer cells. Mol Med Report.

[20] Chen CC, Lee KD, Pai MY, Chu PY, Hsu CC, Chiu CC, Chen LT, Chang JY, Hsiao SH, Leu YW (2015). Changes in DNA methylation are associated with the development of drug resistance in cervical cancer cells. Cancer Cell Int.

[21] Song Y, Zhang C (2009). Hydralazine inhibits human cervical cancer cell growth in vitro in association with APC demethylation and re-expression. Cancer Chemother Pharmacol.

[22] Houseman EA, Accomando WP, Koestler DC, Christensen BC, Marsit CJ, Nelson HH, Wiencke JK, Kelsey KT (2012). DNA methylation arrays as surrogate measures of cell mixture distribution. BMC Bioinformatics.

[23] Viré E, Brenner C, Deplus R, Blanchon L, Fraga M, Didelot C, Morey L, Van Eynde A, Bernard D, Vanderwinden JM, Bollen M, Esteller M, Di Croce L (2006). The Polycomb group protein EZH2 directly controls DNA methylation. Nature.

[24] Brinkman AB, Gu H, Bartels SJ, Zhang Y, Matarese F, Simmer F, Marks H, Bock C, Gnirke A, Meissner A, Stunnenberg HG (2012). Sequential ChIP-bisulfite sequencing enables direct genome-scale investigation of chromatin and DNA methylation cross-talk. Genome Res.

[25] Statham AL, Robinson MD, Song JZ, Coolen MW, Stirzaker C, Clark SJ (2012). Bisulfite sequencing of chromatin immunoprecipitated DNA (BisChIP-seq) directly informs methylation status of histone-modified DNA. Genome Res.

[26] Holland D, Hoppe-Seyler K, Schuller B, Lohrey C, Maroldt J, Dürst M, Hoppe-Seyler F (2008). Activation of the enhancer of zeste homologue 2 gene by the human papillomavirus E7 oncoprotein. Cancer Res.

[27] Rinn JL, Kertesz M, Wang JK, Squazzo SL, Xu X, Brugmann SA, Goodnough LH, Helms JA, Farnham PJ, Segal E, Chang HY (2007). Functional demarcation of active and silent chromatin domains in human HOX loci by noncoding RNAs. Cell.

[28] Sharma S, Mandal P, Sadhukhan T, Roy Chowdhury R, Ranjan Mondal N, Chakravarty B, Chatterjee T, Roy S, Sengupta S (2015). Bridging Links between Long Noncoding RNA HOTAIR and HPV Oncoprotein E7 in Cervical Cancer Pathogenesis. Sci Rep.

[29] Sun NX, Ye C, Zhao Q, Zhang Q, Xu C, Wang SB, Jin ZJ, Sun SH, Wang F, Li W (2014). Long noncoding RNA-EBIC promotes tumor cell invasion by binding to EZH2 and repressing E-cadherin in cervical cancer. PLoS ONE.

[30] Liu Y, Liu T, Bao X, He M, Li L, Yang X (2014). Increased EZH2 expression is associated with proliferation and progression of cervical cancer and indicates a poor prognosis. Int J Gynecol Pathol.

[31] Chen SQ, Zhang HM, Li JB, Jiang HY, Fan L, Kong LZ, Yao SZ (2014). Analyzing simultaneous positive expression of EZH2 and P53 protein to improve predictive value in cervical squamous cell carcinoma. Int J Gynecol Cancer.

[32] Verma SK, Tian X, LaFrance LV, Duquenne C, Suarez DP, Newlander KA, Romeril SP, Burgess JL, Grant SW, Brackley JA, Graves AP, Scherzer DA, Shu A (2012). Identification of Potent, Selective, Cell-Active Inhibitors of the Histone Lysine Methyltransferase EZH2. ACS Med Chem Lett.

[33] Gnyszka A, Jastrzebski Z, Flis S (2013). DNA methyltransferase inhibitors and their emerging role in epigenetic therapy of cancer. Anticancer Res.

[34] Ding M, Zhang H, Li Z, Wang C, Chen J, Shi L, Xu D, Gao Y (2015). The polycomb group protein enhancer of zeste 2 is a novel therapeutic target for cervical cancer. Clin Exp Pharmacol Physiol.

[35] Qu W, Jiang G, Cruz Y, Chang CJ, Ho GY, Klein RS, Burk RD (1997). PCR detection of human papillomavirus: comparison between MY09/MY11 and GP5+/GP6+ primer systems. J Clin Microbiol.

[36] Castle PE, Schiffman M, Gravitt PE, Kendall H, Fishman S, Dong H, Hildesheim A, Herrero R, Bratti MC, Sherman ME, Lorincz A, Schussler JE, Burk RD (2002). Comparisons of HPV DNA detection by MY09/11 PCR methods. J Med Virol.

[37] Clifford GM, Gallus S, Herrero R, Muñoz N, Snijders PJ, Vaccarella S, Anh PT, Ferreccio C, Hieu NT, Matos E, Molano M, Rajkumar R, Ronco G (2005). Worldwide distribution of human papillomavirus types in cytologically normal women in the International Agency for Research on Cancer HPV prevalence surveys: a pooled analysis. The Lancet.

[38] Suzuki M, Jing Q, Lia D, Pascual M, McLellan A, Greally JM (2010). Optimized design and data analysis of tag-based cytosine methylation assays. Genome Biol.

[39] Ulahannan N, Greally JM (2015). Genome-wide assays that identify and quantify modified cytosines in human disease studies. Epigenetics Chromatin.

[40] Jing Q, McLellan A, Greally JM, Suzuki M (2012). Automated computational analysis of genome-wide DNA methylation profiling data from HELP-tagging assays. Methods Mol Biol.

[41] Johnson WE, Li C, Rabinovic A (2007). Adjusting batch effects in microarray expression data using empirical Bayes methods. Biostatistics.

[42] Irizarry RA, Ladd-Acosta C, Wen B, Wu Z, Montano C, Onyango P, Cui H, Gabo K, Rongione M, Webster M, Ji H, Potash JB, Sabunciyan S (2009). The human colon cancer methylome shows similar hypo- and hypermethylation at conserved tissue-specific CpG island shores. Nat Genet.

[43] Subramanian A, Tamayo P, Mootha VK, Mukherjee S, Ebert BL, Gillette MA, Paulovich A, Pomeroy SL, Golub TR, Lander ES, Mesirov JP (2005). Gene set enrichment analysis: a knowledge-based approach for interpreting genome-wide expression profiles. Proc Natl Acad Sci U S A.

