# Supplementary material for: A polycomb-mediated epigenetic field defect precedes invasive cervical carcinoma
Source: Oncotarget. 2016 Aug 19;7(38):62133–43. doi: 10.18632/oncotarget.11390 (PMC5308716; doi:10.18632/oncotarget.11390)
Supplement: Supplementary file 1 [file oncotarget-07-62133-s001.pdf]

A polycomb-mediated epigenetic field defect precedes invasive cervical carcinoma

SUPPLEMENTARY FIGURES AND TABLES

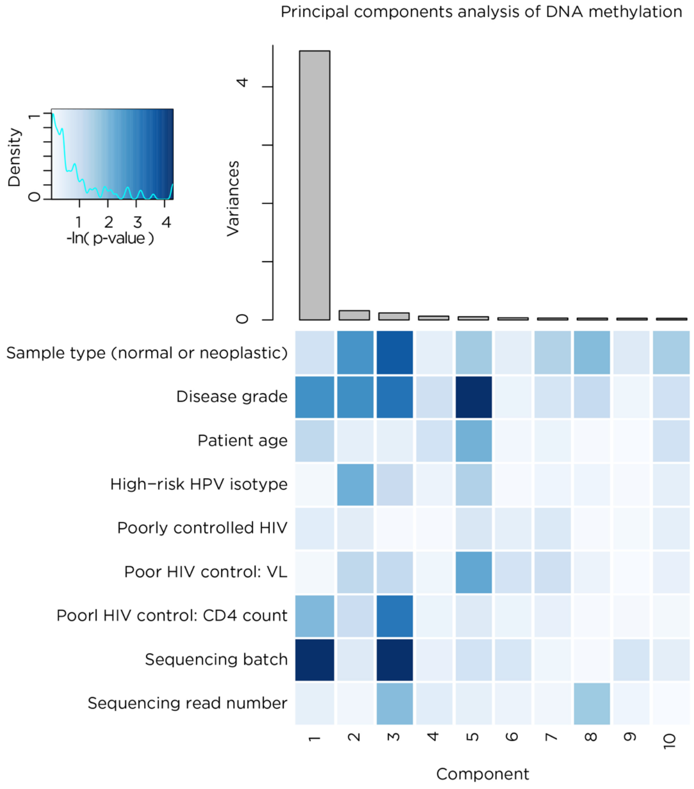

Supplementary Figure S1: Analysis of sources of variability. A principal components analysis was followed by either linear regression for the continuous variables or ANOVA for the categorical variables describing the experiments performed. The influence of each variable is represented by shading depicting the negative natural logarithm p-values for each covariate for each of the first 10 components. We controlled for the effects of the covariates associated with DNA methylation to a greater extent than disease grade, as described in the detailed Methods section above.

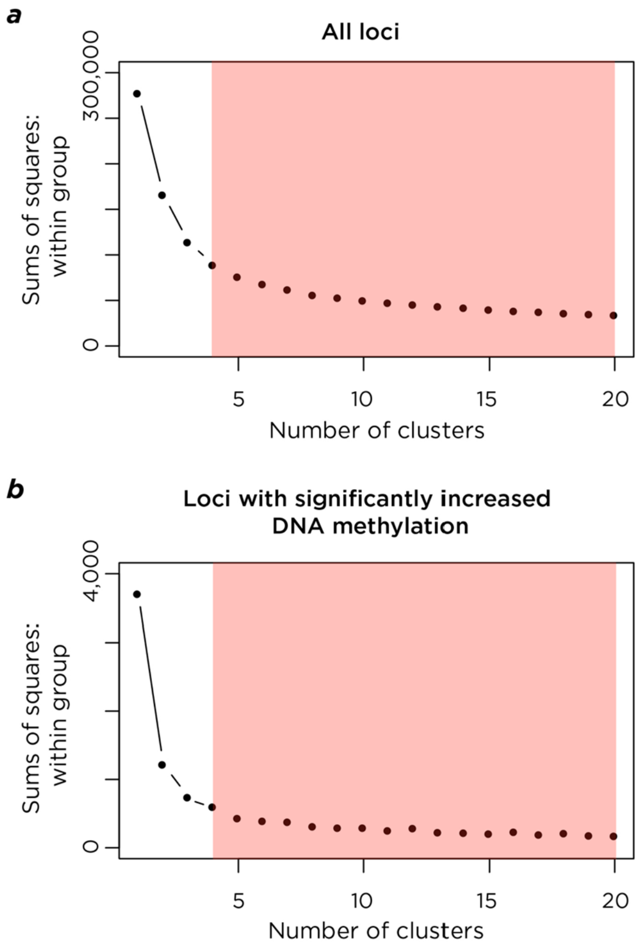

Supplementary Figure S2: Determining the optimal number of k-means clusters for analyses. The within group sums of squares (WSS) is plotted against the number of clusters for **a.** all loci and **b.** the high-confidence subset of loci with significantly increased DNA methylation. The inflection point in the curve is visually estimated and conservative, as shown, interpreted to be the point where additional clusters will over-stratify the data.

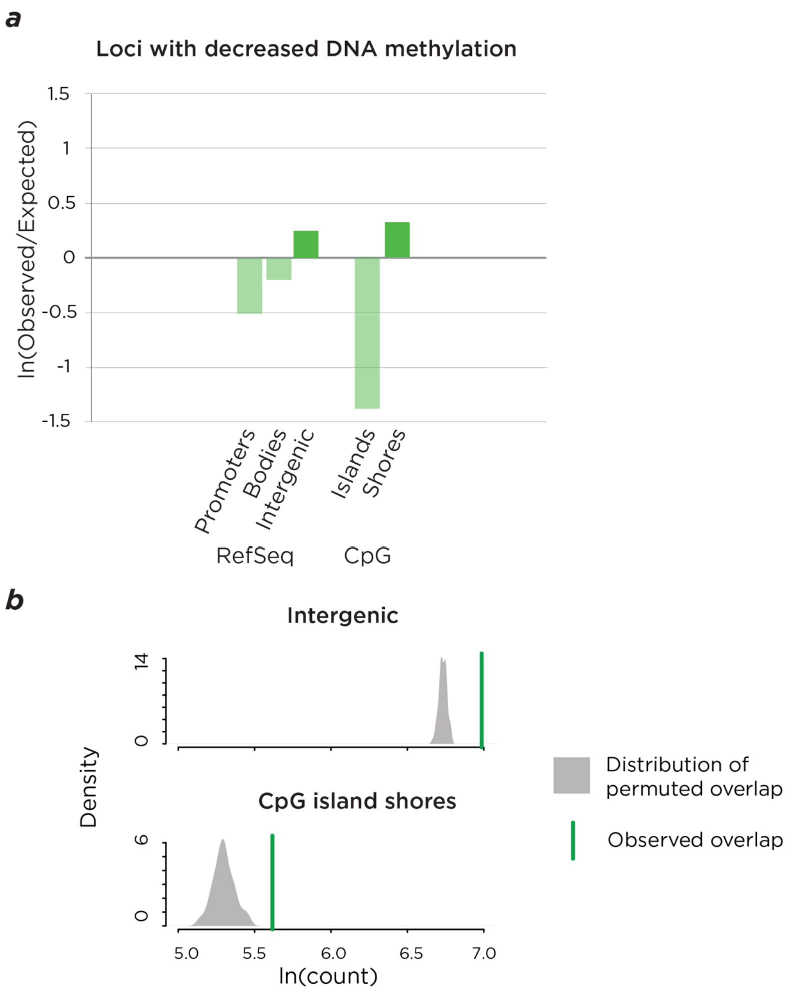

Supplementary Figure S3: Genomic context studies of loci that lose DNA methylation with disease progression. A modest enrichment for intergenic regions and CpG island shores is evidence from the observed/expected ratios of **a.** while **b.** shows the results of permutation testing to reveal that these enrichments are non-random.

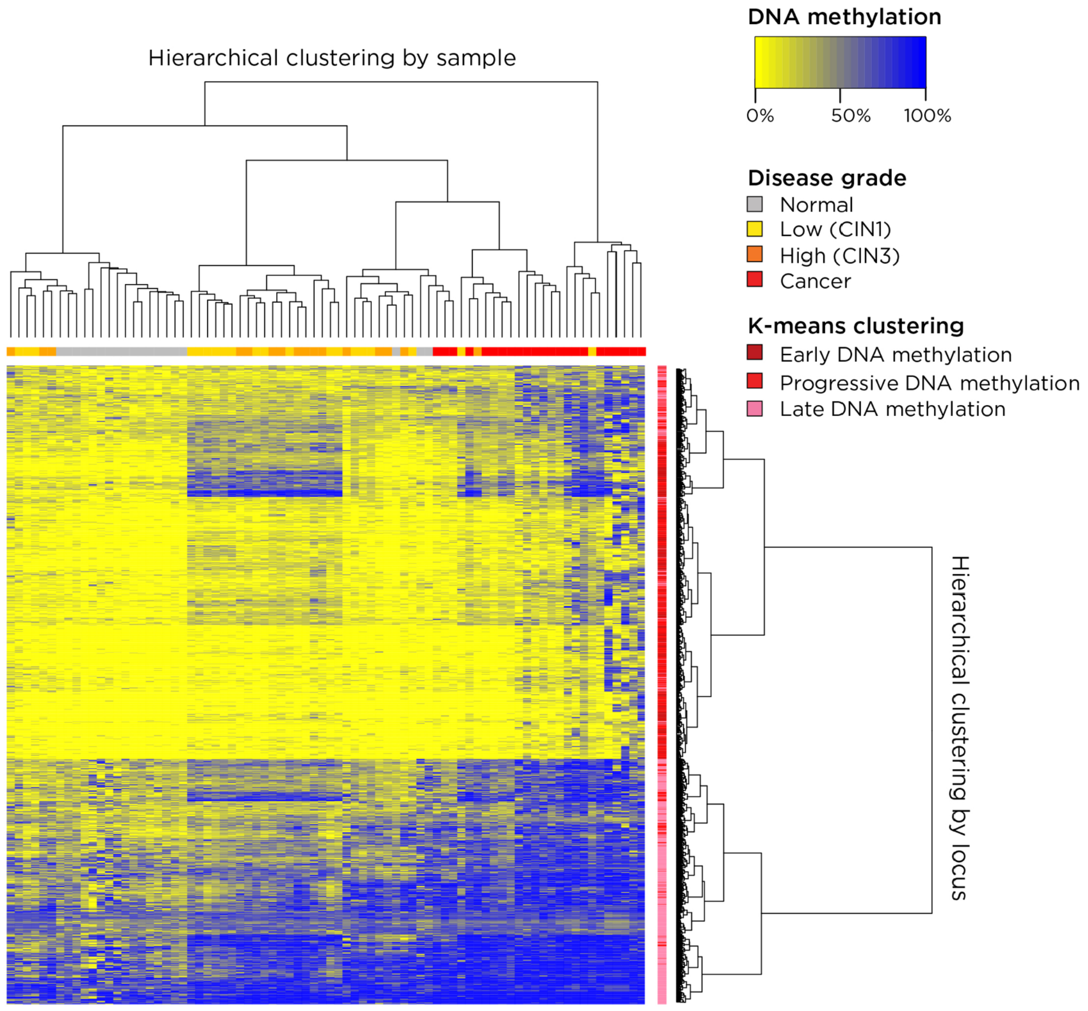

Supplementary Figure S4: A representation combining a heat map of DNA methylation with clustering by sample (columns), using a color code for the disease grades, and clustering by locus (rows), using the same color code of Figure 2 for the loci gaining DNA methylation at early, intermediate or late stages of disease progression.

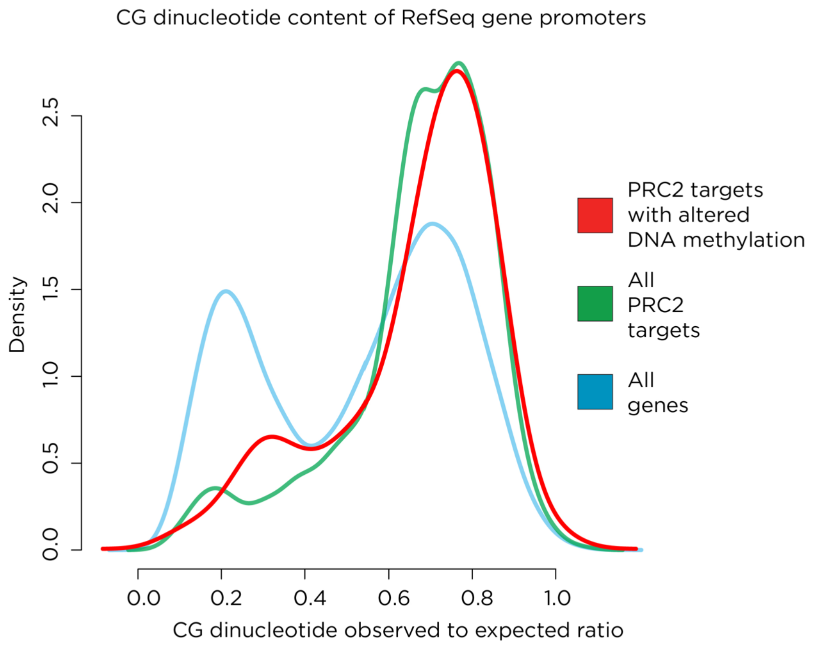

Supplementary Figure S5: CG dinucleotide density analysis of PRC2 target genes. Density plots are shown representing the observed/expected ratios for CG dinucleotides at all genes (blue), all PRC2 target genes (green) and the PRC2 target genes where we found increased DNA methylation at their promoters (red). A strong skewing towards increased CG density is apparent for both groups of PRC2 target genes.

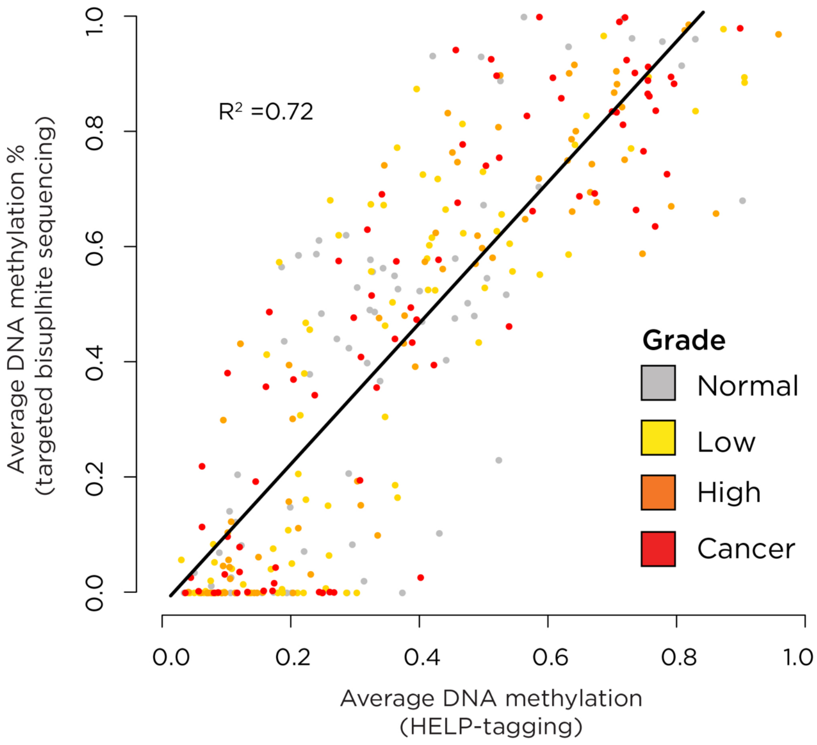

Supplementary Figure S6: Results of targeted bisulphite sequencing. We verified the results of our genome-wide assays using bisulphite PCR followed by sequencing in 84 samples at 70 different loci. The results of DNA methylation calls by the two orthogonal approaches are represented as a scatter plot, showing the expected linear relationship.

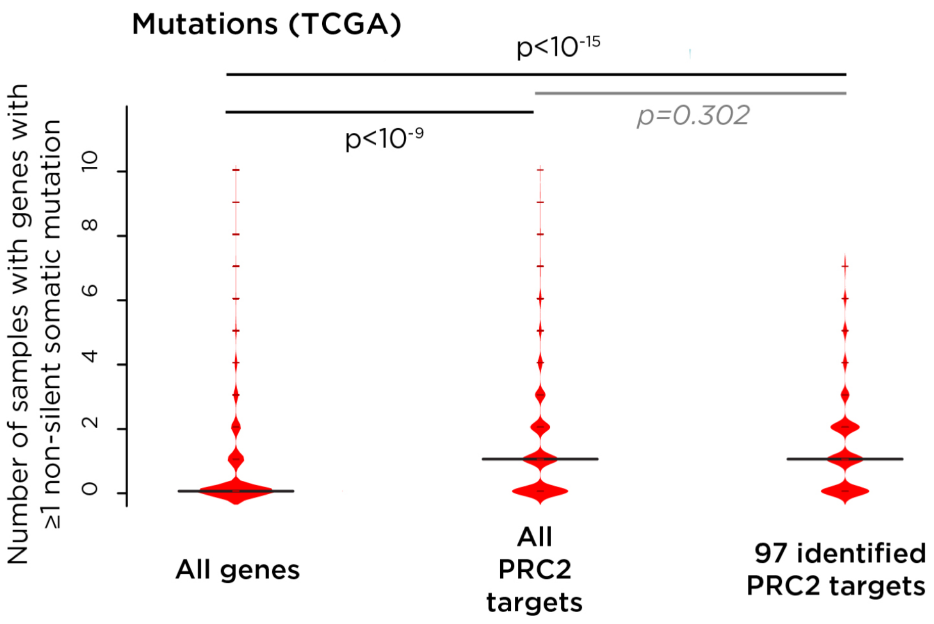

Supplementary Figure S7: Mutation analysis of PRC2 target genes using TCGA data from 183 samples. A y axis value represents the number of samples from the 183 total with mutations in a given gene, while the width of each value represents the proportion of genes in that group with that number of mutations, the black line representing the median. PRC2 target genes are characterized by having an enrichment in mutations compared with the genes throughout the genome.

Supplementary Table S1: Patient and sample characteristics

	Normal		CIN1 (Low)		CIN2/3 (High)		CxCa		*TOTALS*
Number	19		20		16		23		78
Age (SD)	43.7	(6.2)	34.6	(8.0)	36.9	(9.2)	53.3	(14.5)	
African-American	10	53%	8	40%	10	63%	12	52%	*40*
Caucasian	8	42%	10	50%	6	38%	8	35%	*32*
Asian	1	5%	0	0%	0	0%	1	4%	*2*
Unknown	0	0%	2	10%	0	0%	2	9%	*4*
Hispanic	13	68%	12	60%	9	56%	6	26%	*40*
Non-Hispanic	6	32%	8	40%	7	44%	17	74%	*38*
HIV negative	7	37%	13	65%	10	63%	22	96%	*52*
HIV positive	12	63%	7	35%	6	38%	1	4%	*26*
Poor HIV Control*	5	26%	6	30%	4	25%	0	0%	*15*
HPV DNA negative	13	68%	2	10%	3	19%	0	0%	*18*
HPV DNA positive	6	32%	18	90%	13	81%	23	100%	*60*
High-risk HPV positive	1	5%	11	55%	11	69%	23	100%	*46*

*Poor HIV control: any evidence of HIV viremia.

Supplementary Table S2: PRC2 target genes

See Supplementary File 1

Supplementary Table S3: Gene family analysis of PRC2 target genes

Gene	Tumor Suppressors	Oncogenes	Protein Kinases	Homeodomain Proteins	Transcription factors	Cytokines and Growth Factors
BMP6						X
CAMK2B			X			
CMTM2						X
DBX2				X	X	
FGF13						X
FOXB1					X	
FOXD3					X	
GSX1				X	X	
GUCY2D			X			
HLF		X			X	
HOPX				X	X	
HOXC12				X	X	
HOXD4				X	X	
MAPK4			X			
MEOX2				X	X	
MYB		X			X	
NEUROG1					X	
NKX6-2				X	X	
NPAS1					X	
NPAS2					X	
NRG1						X
OLIG1					X	
OTX2				X	X	
PAX1					X	
PAX3		X		X	X	
PAX6				X	X	
PAX7		X		X	X	
PDE4DIP		X				
PITX3				X	X	
RAX				X	X	
SOX21					X	
T					X	
TBX1					X	
TCF21					X	
TLX1		X		X	X	
WT1	X				X	
ZAR1					X	
ZIC1					X	
ZIC4					X	

Supplementary Table S4: Loci for verification studies

See Supplementary File 2
