# Supplementary material for: A polycomb-mediated epigenetic field defect precedes invasive cervical carcinoma
Source: Oncotarget. 2016 Aug 19;7(38):62133–43. doi: 10.18632/oncotarget.11390 (PMC5308716; doi:10.18632/oncotarget.11390)
Supplement: Supplementary file 2 [file oncotarget-07-62133-s002.docx]

**Table S2.** PRC2 target genes.

|  | *Location* | | | *Percent DNA methylation* | | | |
| --- | --- | --- | --- | --- | --- | --- | --- |
| **PRC2_target** | **Chr** | **start** | **stop** | **Control** | **CIN1** | **CIN3** | **CxCa** |
| *ACAN* | chr15 | 89,346,895 | 89,346,896 | 7.48 | 18.90 | 18.48 | 26.66 |
| *ADCY8* | chr8 | 132,052,486 | 132,052,487 | 6.24 | 39.58 | 45.50 | 41.31 |
| *BHLHE23* | chr20 | 61,637,387 | 61,637,388 | 2.65 | 13.18 | 9.68 | 19.34 |
| *BMP6* | chr6 | 7,727,759 | 7,727,760 | 3.40 | 7.07 | 8.30 | 16.49 |
| *CACNA1B* | chr9 | 140,771,910 | 140,771,911 | 3.12 | 5.84 | 5.91 | 17.47 |
| *CACNA1B* | chr9 | 140,772,379 | 140,772,380 | 2.62 | 5.14 | 4.32 | 16.99 |
| *CACNA1B* | chr9 | 140,772,431 | 140,772,432 | 2.63 | 5.04 | 4.68 | 14.31 |
| *CAMK2B* | chr7 | 44,364,665 | 44,364,666 | 2.97 | 27.73 | 34.74 | 27.00 |
| *CBLN4* | chr20 | 54,579,035 | 54,579,036 | 5.46 | 9.33 | 10.59 | 21.03 |
| *CBLN4* | chr20 | 54,579,958 | 54,579,959 | 3.81 | 10.32 | 9.37 | 23.04 |
| *CBLN4* | chr20 | 54,580,030 | 54,580,031 | 5.89 | 10.77 | 12.78 | 21.80 |
| *CCDC140* | chr2 | 223,163,726 | 223,163,727 | 5.59 | 7.31 | 8.26 | 27.07 |
| *CMTM2* | chr16 | 66,612,914 | 66,612,915 | 7.13 | 16.68 | 14.24 | 23.51 |
| *CMTM2* | chr16 | 66,613,237 | 66,613,238 | 8.19 | 17.82 | 16.92 | 26.89 |
| *CNNM1* | chr10 | 101,089,191 | 101,089,192 | 7.37 | 57.55 | 43.22 | 42.50 |
| *COL4A6* | chrX | 107,681,455 | 107,681,456 | 15.25 | 31.44 | 30.99 | 37.81 |
| *COLEC12* | chr18 | 501,396 | 501,397 | 32.82 | 54.50 | 48.21 | 64.15 |
| *CTNND2* | chr5 | 11,903,883 | 11,903,884 | 1.35 | 5.31 | 7.44 | 14.75 |
| *CWH43* | chr4 | 48,988,802 | 48,988,803 | 11.03 | 29.56 | 22.84 | 33.05 |
| *DBX2* | chr12 | 45,444,426 | 45,444,427 | 3.78 | 10.46 | 9.17 | 15.85 |
| *DPP6* | chr7 | 153,585,339 | 153,585,340 | 5.96 | 9.58 | 11.73 | 19.23 |
| *DPP6* | chr7 | 153,585,398 | 153,585,399 | 12.46 | 39.69 | 24.42 | 34.18 |
| *DPP6* | chr7 | 153,749,757 | 153,749,758 | 9.98 | 31.77 | 20.48 | 25.68 |
| *ELAVL3* | chr19 | 11,591,784 | 11,591,785 | 11.68 | 23.49 | 18.56 | 30.28 |
| *FAM19A4* | chr3 | 68,981,498 | 68,981,499 | 10.37 | 20.18 | 16.82 | 26.62 |
| *FBXO39* | chr17 | 6,679,451 | 6,679,452 | 10.40 | 14.39 | 18.98 | 24.57 |
| *FGF13* | chrX | 137,793,921 | 137,793,922 | 6.15 | 9.41 | 8.07 | 18.61 |
| *FIGLA* | chr2 | 71,017,882 | 71,017,883 | 3.77 | 10.07 | 14.54 | 18.88 |
| *FOXB1* | chr15 | 60,296,620 | 60,296,621 | 3.92 | 6.18 | 7.67 | 15.15 |
| *FOXD3* | chr1 | 63,789,665 | 63,789,666 | 7.78 | 23.44 | 20.18 | 28.15 |
| *FOXD4L1* | chr2 | 114,257,596 | 114,257,597 | 15.78 | 35.84 | 31.40 | 37.32 |
| *FOXD4L3* | chr9 | 70,918,633 | 70,918,634 | 15.78 | 35.84 | 31.40 | 37.32 |
| *GFRA1* | chr10 | 118,031,094 | 118,031,095 | 3.71 | 9.32 | 9.07 | 18.66 |
| *GFRA1* | chr10 | 118,032,435 | 118,032,436 | 3.04 | 5.87 | 6.71 | 13.88 |
| *GNA14* | chr9 | 80,262,704 | 80,262,705 | 5.70 | 34.62 | 23.16 | 29.76 |
| *GPC5* | chr13 | 92,051,509 | 92,051,510 | 7.33 | 7.23 | 14.52 | 25.50 |
| *GPR158* | chr10 | 25,464,112 | 25,464,113 | 4.32 | 7.98 | 6.47 | 16.34 |
| *GPR158* | chr10 | 25,465,029 | 25,465,030 | 6.81 | 12.59 | 10.54 | 20.94 |
| *GPR6* | chr6 | 110,300,854 | 110,300,855 | 22.33 | 37.76 | 32.35 | 49.26 |
| *GRIA2* | chr4 | 158,141,527 | 158,141,528 | 15.07 | 31.65 | 33.11 | 36.53 |
| *GRIA2* | chr4 | 158,141,755 | 158,141,756 | 7.42 | 14.51 | 14.53 | 32.13 |
| *GRIN3A* | chr9 | 104,500,304 | 104,500,305 | 2.64 | 13.67 | 7.86 | 13.80 |
| *GSX1* | chr13 | 28,366,822 | 28,366,823 | 6.63 | 20.08 | 13.04 | 30.45 |
| *GUCY2D* | chr17 | 7,906,630 | 7,906,631 | 5.45 | 6.37 | 8.15 | 23.30 |
| *HLF* | chr17 | 53,343,024 | 53,343,025 | 10.29 | 12.34 | 16.73 | 24.35 |
| *HOPX* | chr4 | 57,522,262 | 57,522,263 | 9.65 | 15.28 | 10.73 | 25.90 |
| *HOXC12* | chr12 | 54,349,074 | 54,349,075 | 5.04 | 12.49 | 9.17 | 19.25 |
| *HOXD4* | chr2 | 177,016,563 | 177,016,564 | 4.86 | 7.31 | 10.11 | 18.49 |
| *HS3ST2* | chr16 | 22,825,886 | 22,825,887 | 8.95 | 45.75 | 41.91 | 39.64 |
| *IGFBP5* | chr2 | 217,559,578 | 217,559,579 | 10.51 | 36.25 | 21.62 | 24.14 |
| *KCNA3* | chr1 | 111,217,099 | 111,217,100 | 6.32 | 22.55 | 16.20 | 24.97 |
| *KCNA3* | chr1 | 111,218,083 | 111,218,084 | 14.43 | 10.55 | 13.31 | 31.80 |
| *KCNA5* | chr12 | 5,153,532 | 5,153,533 | 16.94 | 27.38 | 29.39 | 35.01 |
| *KCNIP4* | chr4 | 21,950,351 | 21,950,352 | 4.01 | 8.32 | 8.60 | 17.31 |
| *KCNV1* | chr8 | 110,986,669 | 110,986,670 | 8.23 | 23.08 | 22.51 | 30.24 |
| *LRRTM1* | chr2 | 80,530,750 | 80,530,751 | 16.25 | 36.30 | 41.80 | 44.77 |
| *MAP6* | chr11 | 75,379,621 | 75,379,622 | 12.51 | 28.91 | 20.64 | 29.27 |
| *MAPK4* | chr18 | 48,086,482 | 48,086,483 | 2.67 | 7.29 | 5.60 | 13.00 |
| *MEOX2* | chr7 | 15,726,810 | 15,726,811 | 17.35 | 28.52 | 21.62 | 33.50 |
| *MYB* | chr6 | 135,502,300 | 135,502,301 | 3.72 | 20.90 | 17.93 | 16.44 |
| *NEFH* | chr22 | 29,876,820 | 29,876,821 | 7.34 | 13.76 | 10.93 | 28.42 |
| *NEUROG1* | chr5 | 134,871,152 | 134,871,153 | 5.56 | 17.71 | 11.38 | 22.10 |
| *NEUROG1* | chr5 | 134,871,312 | 134,871,313 | 5.24 | 15.14 | 15.54 | 22.01 |
| *NKX6-2* | chr10 | 134,598,836 | 134,598,837 | 10.83 | 20.26 | 14.87 | 28.84 |
| *NPAS1* | chr19 | 47,524,419 | 47,524,420 | 3.13 | 8.45 | 8.57 | 13.62 |
| *NPAS2* | chr2 | 101,436,389 | 101,436,390 | 3.81 | 38.78 | 21.34 | 16.26 |
| *NPHS2* | chr1 | 179,544,839 | 179,544,840 | 1.99 | 3.99 | 5.24 | 12.61 |
| *NPY5R* | chr4 | 164,265,140 | 164,265,141 | 2.57 | 7.05 | 7.98 | 16.79 |
| *NRG1* | chr8 | 31,497,531 | 31,497,532 | 3.42 | 15.78 | 9.88 | 15.36 |
| *NRG1* | chr8 | 31,498,160 | 31,498,161 | 5.95 | 21.56 | 16.56 | 28.42 |
| *NRN1* | chr6 | 6,007,413 | 6,007,414 | 8.62 | 14.49 | 13.47 | 28.12 |
| *OLIG1* | chr21 | 34,442,985 | 34,442,986 | 6.70 | 12.70 | 9.78 | 19.07 |
| *OTOP3* | chr17 | 72,932,463 | 72,932,464 | 12.21 | 63.52 | 59.16 | 45.71 |
| *OTX2* | chr14 | 57,276,238 | 57,276,239 | 10.84 | 21.78 | 15.21 | 30.31 |
| *OTX2* | chr14 | 57,276,338 | 57,276,339 | 2.62 | 6.39 | 6.07 | 13.89 |
| *PAX1* | chr20 | 21,686,262 | 21,686,263 | 8.92 | 12.31 | 11.78 | 30.04 |
| *PAX3* | chr2 | 223,163,726 | 223,163,727 | 5.59 | 7.31 | 8.26 | 27.07 |
| *PAX6* | chr11 | 31,839,935 | 31,839,936 | 14.26 | 11.06 | 7.26 | 26.43 |
| *PAX7* | chr1 | 18,958,145 | 18,958,146 | 11.30 | 14.57 | 26.74 | 23.57 |
| *PDE10A* | chr6 | 166,075,231 | 166,075,232 | 5.50 | 22.85 | 13.19 | 24.80 |
| *PDE4DIP* | chr1 | 145,075,519 | 145,075,520 | 8.09 | 18.42 | 14.61 | 23.95 |
| *PGM5* | chr9 | 70,972,566 | 70,972,567 | 12.51 | 19.67 | 22.72 | 26.97 |
| *PITX3* | chr10 | 104,001,206 | 104,001,207 | 3.04 | 11.30 | 7.60 | 15.05 |
| *PLA2G7* | chr6 | 46,703,206 | 46,703,207 | 7.89 | 28.59 | 13.75 | 22.60 |
| *PTGDR* | chr14 | 52,734,620 | 52,734,621 | 6.93 | 30.46 | 24.44 | 27.00 |
| *PTGER2* | chr14 | 52,781,835 | 52,781,836 | 10.69 | 22.79 | 22.73 | 32.24 |
| *PTPRT* | chr20 | 41,817,894 | 41,817,895 | 2.99 | 11.07 | 7.41 | 17.00 |
| *RAB6C* | chr2 | 130,737,563 | 130,737,564 | 6.22 | 15.71 | 14.53 | 20.33 |
| *RAB6C* | chr2 | 130,738,213 | 130,738,214 | 40.28 | 66.46 | 60.45 | 70.13 |
| *RAX* | chr18 | 56,939,773 | 56,939,774 | 9.11 | 11.05 | 12.01 | 25.29 |
| *RAX* | chr18 | 56,940,284 | 56,940,285 | 17.37 | 24.36 | 23.01 | 38.34 |
| *RBP7* | chr1 | 10,057,910 | 10,057,911 | 15.40 | 41.07 | 38.52 | 40.72 |
| *SCTR* | chr2 | 120,281,759 | 120,281,760 | 8.61 | 26.74 | 23.15 | 33.76 |
| *SHISA2* | chr13 | 26,624,338 | 26,624,339 | 24.94 | 53.24 | 37.93 | 40.32 |
| *SIAH3* | chr13 | 46,425,495 | 46,425,496 | 32.94 | 35.98 | 28.38 | 50.45 |
| *SLC10A4* | chr4 | 48,485,342 | 48,485,343 | 2.42 | 9.04 | 4.78 | 12.55 |
| *SLC6A11* | chr3 | 10,857,786 | 10,857,787 | 3.82 | 11.34 | 7.49 | 15.92 |
| *SORCS1* | chr10 | 108,923,845 | 108,923,846 | 16.77 | 36.40 | 36.84 | 45.29 |
| *SORCS1* | chr10 | 108,923,845 | 108,923,846 | 16.77 | 36.40 | 36.84 | 45.29 |
| *SORCS1* | chr10 | 108,924,737 | 108,924,738 | 4.60 | 9.50 | 8.45 | 24.21 |
| *SORCS1* | chr10 | 108,925,003 | 108,925,004 | 12.00 | 22.64 | 21.12 | 32.62 |
| *SOX21* | chr13 | 95,365,270 | 95,365,271 | 3.47 | 13.87 | 15.73 | 16.56 |
| *T* | chr6 | 166,581,712 | 166,581,713 | 6.23 | 10.52 | 7.79 | 22.44 |
| *TBX1* | chr22 | 19,743,416 | 19,743,417 | 15.57 | 23.41 | 11.48 | 25.63 |
| *TCF21* | chr6 | 134,211,137 | 134,211,138 | 3.45 | 12.75 | 7.55 | 22.08 |
| *TLX1* | chr10 | 102,891,553 | 102,891,554 | 3.24 | 4.80 | 9.20 | 14.97 |
| *TMEFF2* | chr2 | 193,059,313 | 193,059,314 | 5.32 | 13.14 | 14.15 | 20.55 |
| *TRHDE* | chr12 | 72,666,810 | 72,666,811 | 16.92 | 29.46 | 22.95 | 34.49 |
| *TRHDE* | chr12 | 72,667,151 | 72,667,152 | 9.02 | 15.20 | 14.72 | 31.05 |
| *TTYH1* | chr19 | 54,926,861 | 54,926,862 | 19.24 | 34.00 | 32.08 | 53.90 |
| *UNC5C* | chr4 | 96,470,537 | 96,470,538 | 14.66 | 54.35 | 58.57 | 50.15 |
| *VIPR2* | chr7 | 158,937,481 | 158,937,482 | 4.82 | 19.34 | 14.92 | 18.45 |
| *VSIG2* | chr11 | 124,621,830 | 124,621,831 | 27.74 | 25.32 | 18.21 | 41.10 |
| *VSIG2* | chr11 | 124,621,914 | 124,621,915 | 14.47 | 14.64 | 23.36 | 34.86 |
| *WT1* | chr11 | 32,457,190 | 32,457,191 | 10.87 | 26.40 | 22.33 | 30.62 |
| *WT1-AS* | chr11 | 32,457,190 | 32,457,191 | 10.87 | 26.40 | 22.33 | 30.62 |
| *ZAR1* | chr4 | 48,492,900 | 48,492,901 | 5.44 | 12.29 | 10.93 | 16.16 |
| *ZAR1* | chr4 | 48,493,076 | 48,493,077 | 3.47 | 9.51 | 8.68 | 19.49 |
| *ZAR1* | chr4 | 48,493,241 | 48,493,242 | 9.21 | 16.98 | 16.91 | 30.29 |
| *ZIC1* | chr3 | 147,127,567 | 147,127,568 | 12.56 | 21.67 | 16.52 | 31.63 |
| *ZIC4* | chr3 | 147,111,121 | 147,111,122 | 6.52 | 18.93 | 13.31 | 19.32 |
