# Supplementary material for: A polycomb-mediated epigenetic field defect precedes invasive cervical carcinoma
Source: Oncotarget. 2016 Aug 19;7(38):62133–43. doi: 10.18632/oncotarget.11390 (PMC5308716; doi:10.18632/oncotarget.11390)
Supplement: Supplementary file 3 [file oncotarget-07-62133-s003.docx]

**Table S4.** Loci for verification studies.

| **Target** | **Location** |
| --- | --- |
| *ADAMTS20* | chr12:43945691-43945989 |
| *APOBE3C3* | chr22:39,411,515-39,411,776 |
| *ARMC7* | chr17:73124784-73125030 |
| *CADM1* | chr11:115,247,299-115,247,565 |
| *CDH23* | chr10:73225621-73225903 |
| *CDKN2A* | chr9:21,968,148-21,968,303 |
| *CDKN2A* | chr9:21968781-21968939 |
| *CDKN2A* | chr9:21974469-21974682 |
| *CDKN2A* | chr9:21,968,407-21,968,645 |
| *CDKN2A* | chr9: 21975684- 21975934 |
| *CDKN2A* | chr9:21,970,615-21,970,869 |
| *CDKN2A* | chr9:21993877-21994149 |
| *CDKN2A* | chr9:21994125-21994398 |
| *CDKN2A* | chr9:21,974,893-21,975,181 |
| *CDKN2B* | chr9:22005647-22005865 |
| *CDKN2B* | chr9:22,005,369-22,005,612 |
| *CDKN2B* | chr9:22,008,779-22,009,071 |
| *CMTM1* | chr16:66,612,793-66,613,030 |
| *CNM1* | chr10:101088981-101089241 |
| *CPA1* | chr7:130,019,616-130,019,768 |
| *DAPK* | chr9:90123463-90123664 |
| *DAPK* | chr9:90,113,542-90,113,757 |
| *DAPK* | chr9:90,113,876-90,114,069 |
| *DYNC1I2* | chr2:172,544,447-172,544,752 |
| *FGFR2* | chr10:123,353,272-123,353,530 |
| *GALR1* | chr18:74963962-74964142 |
| *GALR1* | chr18:74963144-74963417 |
| *GATA3* | chr10:8,100,570-8,100,837 |
| *H1C* | chr17:1958090-1958202 |
| *HCP5* | chr6:31,432,447-31,432,669 |
| *HLX* | chr1:221,057,830-221,058,106 |
| *JARID2* | chr6:15,412,002-15,412,146 |
| *KDM4B* | chr19:5,104,049-5,104,334 |
| *KRT32* | chr17:39623347-39623551 |
| *LY6K* | chr8:143,782,172-143,782,415 |
| *MGMT* | chr10:131265717-131265925 |
| *MGMT* | chr10:131321756-131321977 |
| *MGMT* | chr10:131264850-131265134 |
| *MIR4535* | chr22:49,175,026-49,175,258 |
| *NCOR2* | chr12:125,001,183-125,001,346 |
| *NFIX* | chr19:13,108,578-13,108,769 |
| *PAX3* | chr2:223,163,620-223,163,774 |
| *PHACTR3* | chr20:58,190,266-58,190,506 |
| *PHKG1* | chr7:56,150,995-56,151,150 |
| *PTGDR* | chr14:52,734,555-52,734,772 |
| *RAB6C-AS1* | chr2:130738144-130738326 |
| *RASSF1* | chr3:50375273-50375522 |
| *RASSF1* | chr3:50374135-50374405 |
| *RASSF1* | chr3:50378372-50378652 |
| *RPS6KA2* | chr6:166,984,543-166,984,722 |
| *RPS6KA2* | chr6:167,038,658-167,038,907 |
| *SIX6* | chr14:60,977,798-60,977,991 |
| *STX18* | chr4:4,439,712-4,439,945 |
| *TCERG1L* | chr10:133,048,728-133,048,828 |
| *TERT* | chr5:1296076-1296224 |
| *TERT* | chr5:1,293,945-1,294,196 |
| *TERT* | chr5:1,295,772-1,296,048 |
| *TP73* | chr1:3,623,272-3,623,550 |
| *TP73* | chr1:3,626,876-3,627,156 |
| *UNC5C* | chr4:96,470,489-96,470,737 |
| *ZNF469* | chr16:88494369-88494621 |
